# A nomenclature for echinoderm genes

**DOI:** 10.1093/database/baab052

**Published:** 2021-08-13

**Authors:** Thomas R Beatman, Katherine M Buckley, Gregory A Cary, Veronica F Hinman, Charles A Ettensohn

**Affiliations:** Department of Biological Sciences, Carnegie Mellon University, 5000 Forbes Avenue, Pittsburgh, PA 15213, USA; Echinobase, #646 Mellon Institute, 4400 Fifth Ave, Pittsburgh, PA 15213, USA; Department of Biological Sciences, Auburn University, 101 Rouse Life Sciences, Auburn, AL 36849, USA; The Jackson Laboratory, 600 Main Street, Bar Harbor, ME 04609, USA; Department of Biological Sciences, Carnegie Mellon University, 5000 Forbes Avenue, Pittsburgh, PA 15213, USA; Echinobase, #646 Mellon Institute, 4400 Fifth Ave, Pittsburgh, PA 15213, USA; Department of Biological Sciences, Carnegie Mellon University, 5000 Forbes Avenue, Pittsburgh, PA 15213, USA; Echinobase, #646 Mellon Institute, 4400 Fifth Ave, Pittsburgh, PA 15213, USA

## Abstract

Echinoderm embryos and larvae are prominent experimental model systems for studying developmental mechanisms. High-quality, assembled, annotated genome sequences are now available for several echinoderm species, including representatives from most classes. The increased availability of these data necessitates the development of a nomenclature that assigns universally interpretable gene symbols to echinoderm genes to facilitate cross-species comparisons of gene functions, both within echinoderms and across other phyla. This paper describes the implementation of an improved set of echinoderm gene nomenclature guidelines that both communicates meaningful orthology information in protein-coding gene symbols and names and establishes continuity with nomenclatures developed for major vertebrate model organisms, including humans. Differences between the echinoderm gene nomenclature guidelines and vertebrate guidelines are examined and explained. This nomenclature incorporates novel solutions to allow for several types of orthologous relationships, including the single echinoderm genes with multiple vertebrate co-orthologs that result from whole-genome-duplication events. The current version of the Echinoderm Gene Nomenclature Guidelines can be found at https://www.echinobase.org/gene/static/geneNomenclature.jsp

Database URL https://www.echinobase.org/

## Background

Echinoderms have served as important experimental model systems in biology for more than a century, particularly in the field of developmental biology (1–5). Recently, echinoderm embryos and larvae have emerged as a powerful system in which to delineate the gene regulatory networks that operate during embryogenesis (6–9). As a basal lineage of deuterostomes, and because echinoderms exhibit a striking diversity of developmental programs, studies in echinoderms have been invaluable in understanding the evolution of developmental processes (10–16).

Experimental studies using echinoderms have been facilitated, in part, by a wealth of recently available sequencing data. The sequencing of echinoderm genomes began with the Sea Urchin Genome Sequencing Consortium (RRID:SCR_002841) (17), which resulted in genome assembly from the purple sea urchin (*Strongylocentrotus purpuratus*). As technology has advanced, this assembly has been improved several times and is now joined by genome sequences from many other organisms within this phylum. Echinoderm genome assemblies are complemented by a wealth of transcriptome data from several life stages and tissue types (18, 19). These sequence-based resources are a critically important component of modern echinoderm research (20–23). Gene-based information is organized and accessible via Echinobase, the major public repository of echinoderm genomic data (RRID:SCR_013732) (24).

The existing strategy for annotating echinoderm genes involves assigning genes identifiers referred to as ‘names’, rather than ‘gene symbols’, which are more commonly used by other model organism databases (MODs). Proper gene names, which could provide clarification of identity and function, are listed as ‘synonyms’ but are not consistently present for echinoderm genes. As a result, legacy echinoderm gene names are not easily translatable across MODs. This inconsistency raises the risk of researchers incorrectly conflating echinoderm genes with one another or with genes in other organisms based on nomenclature rather than true evolutionary relationships. Establishing coherence in the naming of orthologous genes is a powerful rationale for aligning nomenclatures between different MODs (25). In echinoderms, this problem is further complicated by a lack of clarification and codified standards underlying the present nomenclature. In addition, the high degree of polymorphism in some echinoderm species has led to the existence of numerous artifactual duplicates of individual genes in assemblies (26). Finally, as the amount of sequencing data expands, it becomes increasingly important to ensure that gene symbols and names are both human and machine readable to facilitate their implementation in Echinobase. Standardizing nomenclature according to orthology with established vertebrate genomes will facilitate comparative biological research across deuterostomes.

With the release of an improved, chromosome-scale assembly and annotation of the *S. purpuratus* genome (Spur_5.0), as well as major improvements to Echinobase, the web resource hosting genome-related information, the development of an improved gene nomenclature is timely. Accordingly, an Echinobase Gene Nomenclature Committee (EGNC) was formed to generate a standardized echinoderm nomenclature. The revised nomenclature was developed using the nomenclature guidelines built for *Xenopus* species (27) as a scaffold. A core goal of this nomenclature system is to incorporate robust orthology relationships between echinoderm and human genes when assigning gene symbols and names. Here, we describe these new nomenclature guidelines for echinoderm genes and illustrate their utility in integrating biological information obtained from echinoderms with related information from vertebrate models.

### Criteria for echinoderm genomes

As a product of Echinobase, the nomenclature pipeline has been developed for use with genomes supported by the MOD. Genomes are considered for hosting on Echinobase and processing through our nomenclature pipeline once they have been processed, approved and annotated by NCBI’s RefSeq eukaryotic genome annotation pipeline (28, 29). This serves as an external validator of genome quality and systematically produces provisional gene identifiers for Echinobase. The order of genome integration on Echinobase is then prioritized to improve breadth of taxonomic diversity before expanding depth.

## Nomenclature for echinoderm gene names and symbols

The revised Echinobase gene nomenclature guidelines have transitioned from solely having gene symbols (historically identified on Echinobase as ‘gene names’), to having both full gene names and short symbols (together referred to herein as ‘gene identifiers’). This strategy is modeled after the common features of the nomenclatures used for the human (30), mouse and rat (31), chicken (32), *Xenopus* (33) and zebrafish MODs (34). Furthermore, gene identifiers are formatted similarly to vertebrate gene names and symbols: gene names and symbols are presented in lower case and italicized, and Greek letters and Roman numerals are converted to Latin and Arabic equivalents, respectively (35). As gene pages are updated in accordance with the guidelines described here, all legacy names, symbols and aliases are relegated to the synonym lists for each gene page. When users may wish to refer to a gene by its previous symbol, we recommend referring to it by appending the previous symbol in parentheses following the current symbol. The synonym list is fully integrated into Echinobase’s gene search tool, enabling users to locate genes by historically associated identifiers when needed. Up to date mappings of current gene identifiers to NCBI locus IDs will be provided both through Echinobase’s jbrowse tracks and within Echinobase’s FTP site. While an increasing number of human gene identifiers are being made stable (30, 36), to account for possible changes the up to date gene identifiers of human genes will be annually cross-referenced to keep our nomenclature in alignment.

### Orthology pipeline

To facilitate intra- and interphylum comparisons, echinoderm genes are assigned identifiers on the basis of orthology. To assess orthology, we use the previously described orthology pipeline developed by Echinobase to build orthology maps (37), whose approach is based on the DRSC Integrative Ortholog Prediction Tool (DIOPT) (38) and the HGNC Comparison of Orthology Predictions tool (39), integrating output from several algorithms to build consensus on orthology relationships. Genes are determined to be orthologs if three or more tools used in the Echinobase orthology pipeline support an orthologous relationship, in accordance with Alliance of Genome Resources (RRID:SCR_015850) standards (37, 40). Any orthologous relationships that meet this threshold are used to inform the nomenclature. At present, the orthology pipeline is currently composed of six tools: InParanoid v4.1 (RRID:SCR_006801) (41, 42), ProteinOrtho v6 (43), SwiftOrtho (RRID:SCR_017122) (44), FastOrtho (45), OMA v2.4.1 (RRID:SCR_011978) (46), and OrthoFinder v2.4 (RRID:SCR_017118) (47). Selection of tools is informed by local accessibility/functionality by Echinobase bioinformaticians, resulting in the prioritizing of tools that have publicly available documentation or tools whose providers were able to run our data internally and provide Echinobase with outputs. Anticipated expansion of the orthology pipeline will eventually increase the number of metrics to 12 or more tools to allow for a DIOPT-like analysis (37, 38). The completion of Echinobase’s orthology pipeline with a full suite of tools will precede the implementation of more complex orthologous relationships (e.g. one:many and many:one echinoderm:human orthologs) in our nomenclature as described below.

### Decision regarding nonhuman-vertebrate orthologs and nonvertebrate orthologs

Some consideration was given to expanding the orthology-derived nomenclature to allow for naming echinoderm genes on the basis of orthology with nonhuman orthologs. Three of the orthology tools outputs used in the current Echinobase orthology pipeline are provided from DIOPT [Inparanoid (41, 42), OMA (46) and Orthofinder (47)] that uses protein models derived from the *S. purpuratus* genome and those of numerous major model organisms (37, 38). Of the *S. purpuratus* proteins within this subset of orthology tools that meet the three-criteria threshold (i.e. ‘nameable genes’, 8066 in total; all of these nameable genes were one:one orthologies), 70.79% (5710 proteins) have human orthologs. This reflects 20.8% of all 27 447 *S. purpuratus* protein-coding genes. Further 17.3% of the nameable *S. purpuratus* genes (1392 total; 5.07% of all *S. purpuratus* protein-coding genes) lack orthologs in humans but do have other one:one vertebrate orthologs. For the time being, given this low fraction of genes and the limited coverage of the orthology pipeline at this time, we have maintained the current nomenclature pipeline with a focus on intraphylum and echinoderm:human orthologies; this strategy may be re-visited in the future once the scope of the orthology relationships is expanded for analysis of additional vertebrate species in the Echinobase orthology pipeline tools. *S. purpuratus* genes with only invertebrate or non-animal orthologs comprise 12% of nameable genes from these three tools (964 genes; 3.5% of all *S. purpuratus* protein-coding genes). Because these systems’ nomenclatures include features that are incompatible with ours and those of vertebrate MODs (primarily common use of characters that conflict with our machine-readability criteria), these systems are currently excluded from informing gene nomenclature.

### Nomenclature for echinoderm genes with single human orthologs (one:one)

**Figure 1.**
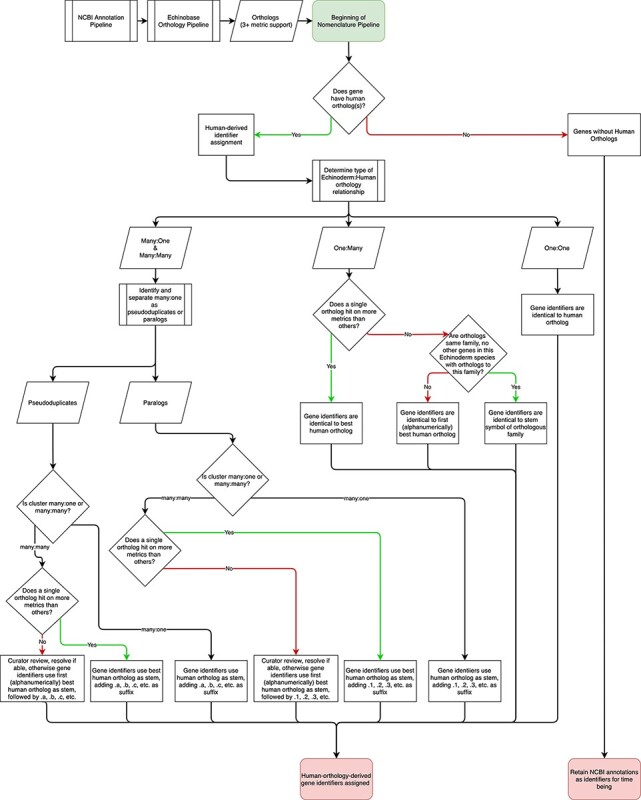
Flowchart describing the Echinobase nomenclature pipeline. Following processing by NCBI and the Echinobase orthology pipeline, gene identifiers are assigned depending on orthology relationships to humans.

The nomenclature pipeline begins with echinoderm genomes processed through the NCBI annotation pipeline, which provides initial annotation. Annotated sequences are then processed through Echinobase’s orthology pipeline (37) and subsequently through the nomenclature pipeline (Figure 1). In the most straightforward cases, the orthology pipeline identifies a single echinoderm gene that is orthologous to a single human gene. If an echinoderm gene is orthologous to a single human ortholog, it is assigned the human gene identifier. The current suite of orthology pipeline outputs is being used to generate one:one orthology-derived gene identifiers, reflecting the low likelihood that further tools will cause shifts in nomenclature but identifiers will be updated in accordance with updates to the orthology pipeline as the need arises. The assignment of more complex orthology relationships between echinoderms and human (i.e. one:many, many:one, and many:many) then go through further processing.

### Nomenclature for echinoderm genes orthologous to multiple human genes (one:many)

Many of the primary differences between echinoderm and vertebrate genome sequences are consequences of the two rounds of whole genome duplication that occurred at the base of the vertebrate clade (48–50). Thus, echinoderm genomes often contain a single gene or smaller subset of genes than are present in vertebrate genomes. This requires a robust set of guidelines for naming echinoderm genes with multiple human orthologs. In the event of multiple orthologs generated by differing numbers of orthology metrics, the approach taken by the EGNC is to assign identifiers to such genes after the ortholog that is supported by the most orthology tools. In the event that multiple human orthologs sharing the same number of metrics are all members of the same gene family (have the same stem symbol) and no members of that family show orthology to another echinoderm species’ gene, the echinoderm gene will be named with the stem, rather than a specific family members identifiers. When this is not the case, curators will examine such sets to determine if relevant information can resolve ties. If this is not possible the first ortholog alphanumerically is selected. This approach is a tradeoff between the loss of orthology information embedded in the gene identifiers and maintaining machine readability and ease of use of identifiers by users. More details of orthology information will be provided on each gene page on Echinobase to supplement informational constraints in the gene identifiers proper.

### Nomenclature for sets of echinoderm paralogs that are orthologous to one or more human orthologs (many:one or many:many)

In contrast to the one:many relationships described above, echinoderm genomes are characterized by many lineage-specific gene family expansions that result in many:one or many:many orthologies with vertebrate genes. This is particularly true for rapidly evolving immune genes and genes involved in making echinoderm skeletons (51, 52). For these cases, genomes are assessed to identify pseudoduplicates, defined here as highly similar genes that may or may not be assembly artifacts, which are treated differently in this nomenclature than paralogs which fall outside of this classification.

## Pseudoduplicates

As echinoderms are broadcast spawners that produce highly outbred diploid crosses, individual echinoderms’ genome sequences are highly polymorphic^817^(53). Consequently, assembling a single haplotype is a challenge and can result in under-collapsed heterozygosity in which several alleles are retained in genome assemblies (27, 54), resulting in groups of genes with highly similar sequences. It is expected that with each new assembly, the occurrence of false duplicates will be reduced and these false expansions will be identified and collapsed. To identify false duplicates, we perform a BLAST of all gene models (including introns and 1 kb up- and downstream of the genic sequence) in each genome against themselves and then extract those that match on 90% or more identity along 90% or more of the longer sequence’s length. This approach is relatively conservative compared to extant methods for identifying false duplicates and collapsing heterozygosity post-assembly (55) and serves to identify genes whose high similarity will confound orthology assignments. Given this likelihood, we refer to clusters of multiple genes with highly similar sequences as ‘pseudoduplicates’.

Genes identified as pseudoduplicates should be assigned the human gene identifiers appended with a decimal point followed by a ‘letter’ (e.g. gene.a, gene.b, etc.). This provides each individual gene with a distinct identity and the associated orthology information while indicating to users that the sequence belongs to a cluster of pseudoduplicates and may require additional confirmation as a true paralog. Suffixes appended to pseudoduplicates in individual echinoderm species will be independent of one another and do not reflect any specific orthology information.

## Paralogs

For sets of paralogous genes that have many:one relationships with humans and are not classified as pseudoduplicates, gene identifiers are matched to the single human ortholog and then appended with a ‘.#’ suffix. This approach conveys both orthology and membership in a gene subfamily. When echinoderm paralogs have multiple human orthologs, the above rule is conjoined with the one:many rules, using whichever human ortholog matches on the most metrics, or, preceding any pertinent determining information, the first alphanumerically if multiple human genes tie for most metrics as the stem for the suffix. Whenever able, orthologs across species will be given the same suffixes.

### Nomenclature for echinoderm genes that lack human orthologs

There remains a set of echinoderm genes (i.e. those without human orthologs) that is not addressed by the preceding components of the nomenclature pipeline. In vertebrate nomenclatures, this is typically addressed by leaving these genes with their NCBI annotation provided identifiers (typically these are formatted with a symbol comprising of LOC followed by the entrez ID of the gene and a name that is based on its predicted protein product), with the opportunity for new identifiers to be generated in conjunction with authors of papers studying such genes (30). This approach is retained here, which allows for the reimplementation of established legacy identifiers provided they meet our nomenclature guidelines, the generation of new identifiers following new studies or an expansion of the Echinobase nomenclature pipeline to additional species (see above), in coordination with the EGNC coordinator.

## Future directions

With assembled genome sequences from *S. purpuratus* and *Acanthaster planci* (OKI-Apl_1.0 (56)) incorporated into Echinobase and assemblies for *Anneissia japonica (ASM1163010v1), Patiria miniata (Pmin_3.0)* and *Lytechinus variegatus (Lvar_3.0* (57)) soon to be supported, intercommunicability of gene identities between species will spur comparative analysis of gene structure and function. ‘Following the assembly of the complete orthology pipeline (12+ tools) more complex (one:many, many:one, many:many) echinoderm:human orthologies will be used to inform associated gene identities’. Planned future additions to the Echinoderm Gene Nomenclature Guidelines include developing nomenclatures for non-coding RNAs, regulatory DNA elements (e.g. enhancers), and gene-related reagents (morpholino antisense oligonucleotides, antibodies, guide RNAs, etc.), in alignment with human gene nomenclature standards whenever possible. As the resources provided by Echinobase expand in conjunction with this revised nomenclature, the usability and functionality of this resource will continue to improve in the coming years.
